# Variability and functional characterization of the *Phakopsora pachyrhizi* Egh16-like effectors

**DOI:** 10.1590/1678-4685-GMB-2023-0192

**Published:** 2024-09-02

**Authors:** Fernanda Machado Castanho, Beatriz Lorena Comin da Costa, Valéria Yukari Abe, Alessandra Yokoyama, Luana Mieko Darben, Liliane Santana Oliveira, Everton Geraldo Capote Ferreira, Ivani de Oliveira Negrão Lopes, Mayra Costa da Cruz Gallo de Carvalho, Maria Isabel Balbi-Peña, Francismar Corrêa Marcelino-Guimarães

**Affiliations:** 1 Centro de Ciências Biológicas, Programa de Pós-Graduação em Genética e Biologia Molecular, Londrina, PR, Brazil.; 2 Centro de Ciências Agrárias, Programa de Pós-Graduação em Agronomia, Londrina, PR, Brazil.; 3 Empresa Brasileira de Pesquisa e Agropecuária (Embrapa Soja), Laboratório de Biotecnologia Vegetal e Bioinformática, Londrina, PR, Brazil.; 4 Departamento de Bioquímica e Biotecnologia, Programa de Pós-Graduação em Biotecnologia, Londrina, PR, Brazil.; 5 Corteva Agriscience, Palmas, TO, Brazil.; 6 2Blades Foundation, Evanston, Illinois, USA.; 7 The Sainsbury Laboratory, Norwich, UK.; 8 Centro de Ciências Agrárias, Programa de Mestrado em Agronomia, Bandeirantes, PR, Brazil.

**Keywords:** Soybean rust, PAMP, effectors, family expansion

## Abstract

Effector proteins in *Phakopsora pachyrhizi* (*Pp*), the causative agent of Asian Soybean rust, are involved in the infection process. A previous study identified a rust effector Egh16-like family based expression profile during the interaction with soybean. Herein, we scrutinized available the *Pp* genomes to validate the predicted Egh16-like family of *Pp* and identify new family members. We described 22 members of the Egh16-like gene family in the *Pp* MT2006 genome and 18 in the UFV02 and K8108 genomes, highlighting a family expansion. Family members have a small signal peptide, conserved cysteine-rich R/Y/FxC motifs in the C-terminal region, and a virulence-related Egh16-like domain and were able to suppress PTI related responses in Benthamiana. Phylogenetic analysis placed the family members into eight clusters, with members induced during the early stages of rust infection. Members of clusters VI and VII are present in different copy numbers in *Pp* genomes and suppressed PAMP-related responses.

## Introduction

*Phakopsora pachyrhizi* (*Pp*) is an obligate biotrophic fungus responsible for Asian soybean rust (ASR) disease. This pathogen has threatened soybean production worldwide and is more severe in countries where the fungus can survive year-round, such as Brazil, Paraguay, and Bolivia. In Brazil, cost with fungicide to control ASR is estimated in $ 2.8 billion per season (Consórcio Antiferrugem, 2019).

Successful infection depends on the secretion of effector proteins that act as virulence factors by manipulating host resistance responses leading to the suppression of PTI (PAMP-triggered immunity). Meanwhile, effectors can act as avirulence (Avr) proteins when host intracellular resistance (R) proteins detect the threat in cooperation with accessory proteins (often the primary Avr ligands), eliciting Effector-Triggered Immunity (ETI) (Kourelis and van der Hoorn, 2018; Chakraborty et al., 2018). PTI is associated with rapid plant response to various pathogens mediated by Pattern Recognition Receptors (PRRs). These are receptor-like kinases (RLKs) or receptor-like proteins (RLPs) with extracellular ligand-binding modules that recognize a particular molecular pattern that confers a broad immune system response. In opposition, ETI acts through direct or indirect recognition of avirulence factors by resistance proteins encoded by plant resistance genes called NLR (Nucleotide-binding leucine-rich repeat) (Jones and Dangl, 2006). Upon recognition, PTI and ETI present different steps in the initial signaling that lead to convergence of several downstream outputs, such as mitogen-activated protein kinase (MAPK) cascades, calcium flux, reactive oxygen species (ROS) burst, transcriptional reprogramming, and phytohormone signaling. These signaling cascades converge and have many intersecting points (Yuan et al., 2021). Both pathways produce reactive oxygen species (ROS), which function as defense and key signaling molecules. PTI induces a rapid and transient ROS burst associated with a biphasic ROS burst. The second peak is usually more intense and long-lasting than the first, leading to cell death, necrosis, and hypersensitivity response (HR). The second peak is race-specific in pathogens that express avirulence proteins (Poltronieri et al., 2020).

Effector proteins acting as avirulence factors often follow a gene-for-gene model. This arms race coevolution affects the genomes of pathogens and hosts, with effectors and resistance genes being the products of the most rapidly evolving families (Toruño et al., 2016). *Pp* variants carrying new virulence alleles can promptly overcome host monogenic resistance. Therefore, genes encoding effectors tend to be found in expanded or lineage-specific families and encode cysteine-rich and small secreted proteins (SSPs) that are frequently secreted from the haustorium during the biotrophic phase of rust infection (Dodds et al., 2004; Catanzariti et al., 2006; Rafiqi et al., 2010). However, the expression of effectors can occur in spores and during the initial steps of host interaction, before haustorium formation (Nirmala et al., 2010) or throughout the entire infection process (Duplessis et al., 2011 a). These proteins are secreted into the apoplast or taken up by the cytoplasm of host cells. The first effectors characterized for rust fungi were proteins secreted from the haustorium for uptake into host cells (Dodds *et al.*, 2004; Kemen et al., 2005). The products of several avirulence genes in the flax rust fungus *Melampsora lini* are recognized by resistance proteins inside the plant cells (Duplessis *et al.*, 2011b). 

Functional characterization of rust effectors can be achieved through *in silico* and *in vitro* analyses and by heterologous *in vivo* experiments (Duplessis et al., 2011a; Lowe and Howlett, 2012; De Carvalho et al., 2017). Recently, the prediction of potential effectors in pathogens causing rust in wheat, poplar, and ASR allowed the screening of immunosuppressive functions taking advantage of surrogate expression systems coupled with *Nicotiana benthamiana* as a substitute for natural hosts (Petre et al., 2015; Kunjeti et al., 2016; Liu et al., 2016; Qi et al., 2016; Cheng et al., 2017; Dagvadorj et al., 2017; De Carvalho *et al.*, 2017; Ramachandran et al., 2017). Using this strategy, Kunjeti *et al.* (2016) agroinfiltrated constructs expressing four secreted effector protein candidates in *N. benthamiana* leaves and found that two of these proteins promoted the virulence of *Phytophthora infestans*. Qi *et al.* (2018) evaluated a set of 82 haustorially-expressed proteins from *P. pachyrhizi* described as potential effector families by Link et al. (2014). They found 17 proteins capable of suppressing PTI and one (*Pp*EC23) able to suppress ETI. De Carvalho *et al.* (2017) described the *Pp* secretome based on 851 predicted proteins, highlighting 13 gene families, three of them containing members presenting the most known effector features (Families 1, 2, and 3), such as small protein size (<200 amino acids), enrichment of cysteine residues, the presence of an N-terminal secretion signal, and the presence of motifs that could direct fungal effectors to particular subcellular compartments in plant. Members of Family 1 (denovo_1784 and denovo_2238) were able to suppress the HR in *N. benthamiana* infiltrated with Pst DC3000, indicating their potential role in ETI (De Carvalho *et al.*, 2017). To date, denovo_1748, denovo_2238 (Family 1), and *Pp*EC23 (Qi *et al.*, 2018) are the only secreted proteins of *Pp* with demonstrated ETI suppression ability. 

Recently, the genome of *Pp* was sequenced from three isolates (Gupta et al., 2023), open new alternatives to genomic studies (Chicowski et al. 2024), and providing information on the entire gene arsenal that characterizes this phytopathogen (https://mycocosm.jgi.doe.gov/Phakopsora/Phakopsora.info.html). From the genome data, we compared the transcripts previously predicted as representatives of Family 1 (De Carvalho et al., 2017), which allowed the characterization of the whole family. These data combined with large-scale expression and phylogenetic studies may identify members with relevant roles in the (a)virulence and coevolution of the pathosystem.

In the present study, we characterized the Family 1 of *Pp* as comprising 22, 18 and 18 members in the genomes of MT2006, UFV02, and K8108, respectively. We demonstrated a conserved structural pattern in the family: the presence of the Egh16-like domain DUF3129/PFAM11327 in all sequences, that is, characteristics of proteins widely encoded in the genomes of phytopathogenic fungi and associated with appressorium formation. Furthermore, the expression profile, and the ability to suppress PTI during infection for some members were also presented, highlighting evidence that these effectors with Egh16-lig domain may be essential for virulence in *Pp* and potentially part of the core effectors in this species.

## Material and Methods

### Characterization of members of the Pp Egh16-like family based on genome annotation

To verify the corresponding *Pp* gene models of the Family 1 members predicted (De Carvalho et al., 2017), we performed BLAST X, N, and P searches on the available genomes of the isolates MT2006, UFV02, and K8108 using an e-value cutoff of 1e^-5^. We selected the best hit with at least 70% identity and 90% coverage for each sequence using Blast P (Table S1). To guarantee the identification of complete gene models, these models were also BLASTed against members of Family 1, using the same cutoff.

We used the MCL cluster analysis from the JGI website (available at https://mycocosm.jgi.doe.gov/Phakopsora/Phakopsora.info.html) to select new potential gene models in Family 1 in the *Pp* genome assembly. In this analysis, we used sequences returned from the BLASTP searches against *Pp* genomes MT2006, UFV02, and K8108 (Table S2) and the genomic sequences of nine rust species, *Cronartium quercuum f.sp. fusiforme* v1.0, *Melampsora americana* v1.0, *Melampsora lini* CH5, *Melampsora larici-populina* v2.0, *Melampsora medusae* f.sp. *deltoidae* v1.0, *Puccinia graminis* f.sp. *tritici* v2.0, *Puccinia triticina* 1-1 BBBD Race 1, *Puccinia striiformis* f.sp. *tritici* PST-130, *Puccinia striiformis* f.sp. *tritici* 104 E137A.

Protein sequences selected in the previous steps were subjected to similarity searches using the hmmsearch program (HMMER package) (Eddy, 2011) against Pfam models (Mistry et al., 2021). We used default parameters to identify the occurrence of protein domains in the sequences. Subsequently, the SignalP5 program (Armenteros et al., 2019), with default parameters, was used to determine the presence of signal peptides in the sequences. Finally, we applied an in-house Perl script to identify the number of cysteine residues in each protein sequence and to search for motifs in the proteins predicted from the gene models. The motifs CSFY, CXY, FXC, RCR, RXLR, SIIR, WXC, WXL, and YXC were searched for in the sequences. All these motifs, except RCR, were selected because they have already been described in the literature as being present in oomycete or rust fungal effector proteins (Kale, 2012; Sperschneider et al., 2015; Liu et al., 2019; Zhao et al., 2020). The RCR motif was selected because of its identification in the sequences of Family 1 members (De Carvalho et al., 2017).

### Phylogenetic tree and structural analysis of Pp Egh16-like Effectors

**Figure 1-:**
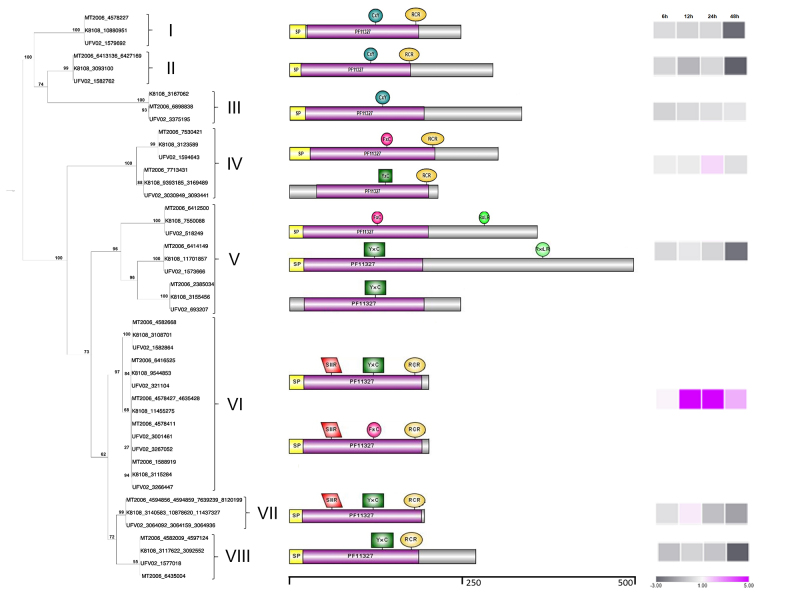
Phylogenetic tree from protein sequences and protein structure for the 58 redundant gene models of Family 1 members from MT2006, UFV02, and K8108 *P. pachyrhizi* genomes and expression profile. Redundant gene models with the same protein length and 100% identity, and 100% coverage in the nucleotide sequence in the same genome appear in the same line in the tree. The structures of the proteins referring to each cluster next to the tree represent the signal peptide, the purple bar PF11327 represents the Egh16-like domain, and the conserved motifs show their positions in each protein. On the right, a graphical representation of the expression for eight *P. pachyrhizi* effector candidates during soybean infection. The relative gene expression levels obtained using RT-qPCR from infected soybean leaves are represented in blocks, from left to right, collected at 6, 12, 24, and 48 h after inoculation (hai).

To correlate the members of the transcriptome data, we constructed an initial phylogenetic tree based on multiple sequence alignments (MSA) between gene models from the MT2006 genome (Figure S1). For the second phylogenetic tree construction and structural analysis, we used protein sequences from the gene models predicted in the three genome assemblies from the *Pp* isolates MT2006, UFV02, and K8108 (Figure 1). Initially, we manually corrected sequences that showed errors, such as sequences presenting a late start codon or errors in exon-intron boundaries. The resulting MSAs obtained in the MUSCLE program (Edgar, 2004) were used to build phylogenetic trees with the IQ-TREE program (Minh et al., 2020), both using default parameters. To edit the images of the trees, we used the Dendroscope program (Huson and Scornavacca, 2012).

The structures of the representative effector proteins were drawn based on the shared characteristics of the members in each branch of the phylogenetic tree using the web application Illustrator for Biological Sequences (IBS) as the illustrator for presentation and visualization of biological sequences (http://ibs.biocuckoo. org/online.php).

### Expression profile of Pp Egh16-like members

At least one member in each cluster formed in the phylogenetic tree of Family 1 was selected to validate the expression profiles of the effector candidates. Six candidates were selected (MT4578227, corresponding to denovo_2595 - cluster I; MT6413136 and MT6427169 corresponding to denovo_251 - cluster II; MT6898838, corresponding to denovo_635 - cluster III; MT4635428/ MT4578427/ MT4578411/ MT4582668/ MT6416525/ MT7530421/ MT7713431 and MT1588919, corresponding to denovo_1784 - cluster VI; MT4582009/ MT4597124/ MT6435004, corresponding to denovo_555 - cluster VIII and MT8120199/ MT7639239/ MT4594856 and MT4594859 corresponding to denovo_2238 - cluster VII) jointly with two candidates only predicted in the genome, MT2006_6414149 and MT2006_7713431. We quantified the expression levels at early time points after inoculation (0, 6, 12, 24, and 48 h) of leaves of the susceptible BRS 184 cultivar with Brazilian Pp isolate CMES 1962. Each experiment comprised three biological replicates, each containing three plants. RNA extraction was performed using 100 mg of frozen leaf tissue in 1 mL of TRIzol reagent (Invitrogen). RNA was quantified using Nanodrop (Uniscience), and then 1 μg of RNA was treated with RNase-free DNase (Invitrogen). cDNA synthesis used the SuperScript III First Strand kit (Invitrogen). We performed the RT-qPCR analysis utilizing primer designed based on transcriptome sequences and gene models. RT-qPCR was performed on a 7900HT Fast Real-Time PCR System (Applied Biosystems) using SYBR green chemistry. We quantified samples in two independent runs and technical triplicates. The expression levels were determined using relative quantification, calculated using the software REST^©^ (Pfaffl et al., 2002). The *Pp* CytB gene served as an internal normalizer (Link et al., 2014) and time zero as the calibrator in the relative quantification analysis. The data were log-transformed to generate heat maps using Morpheus software (https://software.broadinstitute.org/morpheus/).

### Functional characterization of Pp Egh16-like effector candidates

Potential effectors of *Pp* Family 1 MT8120199/ MT7639239/ MT4594856/ MT4594859 (corresponding to denovo_2238/cluster VII) and MT4635428/ MT4578427/ MT4578411/ MT4582668/ MT6416525/ MT7530421/ MT771343/ MT1588919 (corresponding to denovo_1784/cluster VI) were selected for functional characterization because of their high similarities with gene models predicted in the *Pp* genome and their ability to suppress ETI (De Carvalho et al., 2017). The genes were evaluated for their ability to suppress PTI and their expression levels throughout the infection cycle after innoculation with the two monosporic isolates.

*N. benthamiana* plants were grown in a growth chamber at 24 °C with a 12 h photoperiod. The effector candidates were cloned into *Pseudomonas fluorescens* Pf0-1 containing the hrp genes responsible for allowing the translocation of proteins from these bacteria, known as the Effector-to-Host Analyzer (EtHAn) (Thomas et al., 2009; Gimenez-Ibanez et al, 2018), using the pEDV6 plasmid (Sohn et al., 2007). Recombinant EtHAn bacteria were grown in King’s B (KB) medium with chloramphenicol 30 µg/mL and gentamicin 50 µg/mL for 48 h at 28 ºC (King *et al.*, 1954; Thomas *et al.*, 2009). The bacterial colonies were resuspended in water and centrifuged at 5000 rpm for 15 min, and the pellets were resuspended in 2 mL of infiltration buffer (MgCl2 10.0 mM) and adjusted to an OD600 of 0.5.

The abaxial side of leaves of five-week-old *N. benthamiana* plants were infiltrated with bacterial suspensions using a needleless syringe. As a control, *N. benthamiana* leaves were infiltrated with a buffer solution and bacteria containing the pEDV6 empty vector. The controlled factors in the experiment consisted of effector treatments and nine replicates in a randomized experimental design.

*N. benthamiana* infiltered leaves were used for ROS detection and callose deposition analysis. For ROS detection, leaf discs were collected 24 h post infiltration and stained overnight with diaminobenzidine (DAB) with slight stirring at room temperature. The leaf discs were then washed with distilled water, destained for 15 min with a warm ethanol solution: glycerol: acetic acid (3:1:1), and mounted on 50% glycerol. The samples were analyzed using an optical microscope, and pictures were taken using Motic^®^ Images Plus 2.0 software.

Foliar discs were collected 72 h post infiltration to quantify callose deposition in *N. benthamiana* leaves. The foliar discs were destained overnight in 95% ethanol with agitation at 37 °C, followed by several successive washes with 70% or 30% ethanol and water. The foliar discs were stained with a solution of aniline blue (150 mM potassium phosphate, pH 9.0) for 1.0 h under agitation in the dark at room temperature. The samples were examined using a fluorescence microscope with a DAPI filter, and the images were analyzed using Zeiss AxioVision 3.0.

ROS quantification and callose deposition was performed by quantifying the pixels using a negative control (buffer) as a calibrator. Statistical analysis was performed using the t-test, comparing the positive control mean (pEDV6 empty vector) with the pEDV6 recombinant using a p-value of 0.01. 

## Results

### Characterization of Pp Family 1 effectors

To identify the corresponding gene models (Table 1), the 22 transcripts identified in Family 1 by a previous transcriptome study (De Carvalho et al., 2017) were searched in the three available *Pp* genomes (Gupta et al., 2023). Based on the criteria established for the BLAST similarity assumption, only eight predicted transcripts were mapped to the gene models in each *Pp* genome (Table S1). The non-validated transcripts were likely assembly errors, considering that the reference genomes were unavailable at the time of transcriptome assembly. Manual inspection of Family 1 transcripts and correlated gene models showed that the transcripts denovo_5381 and denovo_5849 represented erroneous assemblies of the transcript denovo_2238 and were, therefore, excluded from the analysis. In contrast, the transcripts denovo_6062, denovo_3838, and denovo_7507 were mapped onto erroneously assembled gene models, such as MT2006_452009/MT2006_4597124, and were excluded from the family.

**Table 1-:** Characterization of Pp Family 1 gene models in the MT2006, PPUFV02, and K8108 genomes.

Protein ID/ Gene Models corresponding transcript (*de novo*)				SP	Protein length (aa)	% Cys	MCL Cluster	Pp Family 1 Cluster
MT2006	PPUFV02	K8108	*De novo*					
4578227	1579692	10880951	1303 2595	1-24	247	2,4	125	I
6413136 6427169	1582762	3093100	251	1-20	306	1,9	1253	II
6898838	3375195	3167062	635	no	289	2,8	2780	III
7530421	1594643	3123589	1784	1-30	277	2,1	125	IV
7713431	3030949 3093441	9393185 3169489	1784	no / 1-28	205-239	3,4^†^	125	
6412500	518249	7550088	2238 1402	1-20	348	2,0	125	V
6414149	1573666	11701857	555 1770 2227	1-24	500	1,2	125	
2385034	693207	3155456	1170 2227	no	247	2,4	125	
4582668 6416525 4578427 4635428 4578411 1588919	*1582864* *321104* *3001461* *3267052* 3266447	3108701 9544853 11455275 3115284	1784	1-22/24	202 - 204	3,2^†^	125	VI
8120199 7639239 4594856 4594859	*3064936* *3064159* *3064092*	3140583 11437327 10878620	2238 1402	1-23	203	3,4	125	VII
4582009 4597124 6435004	*1577018*	3117622 3092552	555 1770 2227	1-20	195 - 257	3,0^†^		VIII

SP: signal peptide; Cys: Cysteine.

^†^ average for genetic models with variations in the number of Cysteines.

Gene models in italic in PPUFV02 belongs to expanded families by Gupta et al., 2023.

To identify additional sequences in the *Pp* genomes that shared similarities with Family 1 members, we searched for MCL gene clusters in JGI (Table S1). This search identified 14 additional gene models in the MT2006 genome, and 10 in each of the UFV02 and K8108 genomes. Therefore, considering both strategies, we identified 22 gene models in the MT2006 genome and 18 in the other two genomes. 

We observed duplications in some unique gene models in the three genomes (Table 1, Table S1). This was detected for MT2006_6413136 and MT2006_6427169, similar to denovo_251, with identical protein sequences, but positioned in distinct genome regions. Likewise, the denovo_2238/denovo_1402 transcripts were analogous to the four gene models in the MT2006 genome (MT2006_ 4594856, MT2006_ 4594859, MT2006_ 7639239, and MT2006_ 8120199) and the three gene models in the other genomes (UFV02_ 3064092/UFV02_ 3064159/UFV02_ 3064936 and K8108_3140583/K8108_10878620/K8108_ 11437327), all of which encode identical proteins. 

The MT2006_4578427 and MT2006_4635428 gene models, similarly to the denovo_1784 model, also had identical protein sequences mapped in different regions in the genome. This transcript was similar to four other gene models in the MT2006 genome, five in the UFV02, and four in the K8108 genome. However, the proteins coded from these gene models were not identical (>90% identity). In MT2006 genome: MT2006_4578411, MT2006_4582668, MT2006_6416525 and MT2006_1588919 were similar to denovo_1784, the gene models UFV02_3267052, UFV02_3266447, UFV02_3001461, UFV02_1582864, and UFV02_321104 in the UFV02 genome and K8108_ 9544853, K8108_ 3108701, K8108_ 3115284 and K8018_ 11455275 in the K8018 genome (Table S1). 

### Pp Egh16-like virulence effectors are highly conserved in the Pp genomes

A phylogenetic tree was generated using all 58 gene models initially identified from the three *Pp* genomes. The tree resulted in the formation of eight clusters, each one being composed of at least one gene model from each *Pp* genome (Figure 1). Clusters I and III were the only clusters represented by a single-gene model for each genome. In contrast, Cluster VI was the most represented in the tree, with 27 members: 10 in the UFV02 genome, nine in MT2006, and eight in the K8108 genome. 

Searches for conserved regions were performed in 45 non-redundant Family 1 gene models and their corresponding transcripts, which showed that the Egh16-like domain (PFAM11327) of *Erysiphe graminis* f. sp. *hordei* virulence factor family (Xue et al., 2002) occurred in all family members. We also found high conservation for the motifs RCR, YxC, and SIIR, with frequencies of 80.3%, 54.5%, and 51.5 %, respectively (Table 2).

**Table 2-:** Percentage of the conserved motifs identified in Family 1/Egh16-like members of Pp genomes (MT2006, UFV02 and K8108), and transcript sequences (*de novo*).

Motifs	% of sequences				
	MT2006	UFV02	K8108	*De novo*	Mean
CXY	18.2	16.7	16.7	50	21.2
FXC	27.3	27.8	22.2	12.5	24.2
RCR	81.8	77.8	77.8	87.5	80.3
RXLR	9.1	11.1	11.1	0	9.1
SIIR	54.5	50.0	50.0	50	51.5
YXC	54.5	55.6	61.1	37.5	54.5

The RCR motif was not observed in Clusters II and V members. However, Clusters VI and VII members showed a conserved structure of the SIIR, YxC, and RCR motifs. Clusters VI and VII were composed of gene models MT4635428/ MT4578427/ MT4578411/ MT4582668/ MT6416525/ MT7530421/ MT7713431 and MT1588919, corresponding to denovo_1784, and MT8120199/ MT7639239/ MT4594856 and MT4594859, corresponding to denovo2238 transcripts, and were present in three or more copies in *Pp* genomes. Both denovo_1784 and denovo_2238 were significantly induced during infection, with a peak induction at 12 hpi (Table S3, Figure 1). Except for the members of Cluster III (denovo_635), all the members analyzed during rust infection showed a decline in expression at 48 hpi compared to 24 hpi, indicating a probable relationship between these genes and early rust infection processes.

### Effector members of Egh-16 like, in Pp, can suppress PAMPs-related responses

**Figure 2-:**
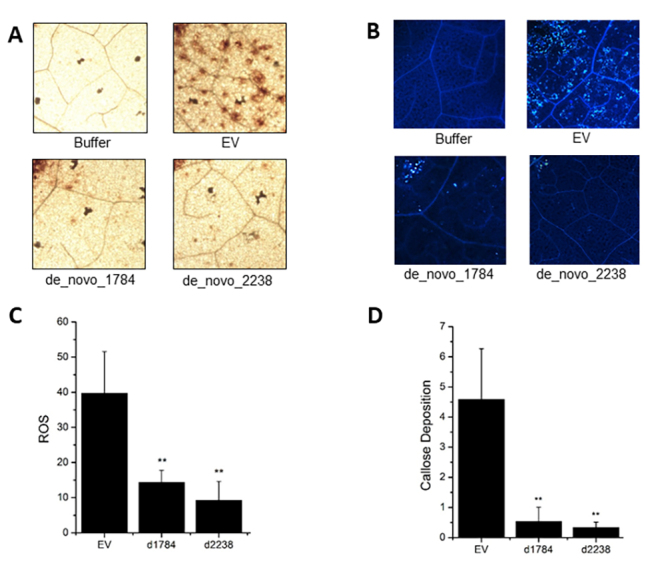
Suppression of ROS production and callose deposition. (A) DAB pigmentation for ROS production detected on *N. benthamiana* leaves (10×). (B) Callose deposition stained with aniline blue in *N. benthamiana* leaves (5×). (C) ROS quantification. (D) Quantification of callose deposition. Buffer: negative control; EV: Empty Vector, positive control; effector proteins: denovo_1784 and denovo_2238.

ROS production and callose deposition are relevant markers of the basal defense responses to pathogen infection. These responses were observed when EtHAn was inoculated into an empty vector (Figure 2). However, the inoculation of EtHAn containing the gene models MT8120199/ MT7639239/ MT4594856/ MT4594859 (corresponding to denovodenovo_2238/cluster VII) and MT4635428/ MT4578427/ MT4578411/ MT4582668/ MT6416525/ MT7530421/ MT771343/ MT1588919 (corresponding to denovodenovo_1784/cluster VI) showed a reduction in ROS pigmentation and callose deposition in levels similar to that observed for the negative control (buffer solution of 10mM MgCl2), indicating that these effectors suppress efficiently the PAMP-related responses. This reduction was significant compared to infiltration with the empty vector (t-test, p < 0.01).

## Discussion

Despite the importance of the ASR on soybean production, the identification and functional characterization of *Pp* effectors remains largely incomplete (Kunjeti et al., 2016; Qi et al., 2018). Link et al. (2014) first identified a set of transcripts as potential haustorial *Pp* effectors. Later, a list of 851 sequences was reported as the potential secretome of *Pp,* among which transcripts grouped into different families, including Family 1 (22 transcripts), were suggested as effector candidates (De Carvalho et al., 2017). Indirect evidence of effector functions has been reported for some of these genes. These results were achieved using transcriptomic strategies without a *Pp* reference genome. Therefore, because of the public availability of the *Pp* genome (Gupta et al., 2023), it has become possible to search for effector EST candidates in the genome and to predict all the family members based on the evolutionary aspects of family expansion and conservation. 

Analyses comparing the transcriptome from Family 1 predicted by De Carvalho et al. (2017) and the MT2006, UFV02, and K8108 genomes allowed us to identify 22 gene models in the MT2006 genome, corresponding to eight previously identified transcripts and 18 gene models that were common to the UFV02 and K8108 genomes (Table S1). Some *Pp* transcripts were shown to be redundant, representing errors in transcriptome predictions. However, others may still represent alternative copies since the transcriptome study was conducted using a Brazilian population of *Pp* from Londrina fields collected in 2013 and was not represented among the isolates used in the genome assemblies.

The analysis of motifs showed the conservation of the [FY] xC motif among Family 1 clusters. The [FY]xC motif has been described in studies involving proteins from *M. larici-populina*, *P. graminis* f. sp. *tritici,* and *Blumeria graminis* f. sp. *hordei* (Godfrey et al., 2010; Pedersen et al., 2012; Saunders et al., 2012). They may play a role in protein folding (Pedersen *et al.*, 2012). Other motifs, such as RCR and SIIR, were detected in protein sequences from different clades in the phylogenetic tree. RxLR, a well-known motif in oomycete effectors involved in the translocation of proteins to the host cell (Whisson et al., 2007), was only detected in the member MT2006_6412500 and its correspondents K8108_7550088 and UFV02_518249 in the other two genomes.

The prediction of Egh 16-like members in the *Pp* genomes resulted in the Egh16-like domain identification in all members with a 100% match (Figure 1). Some of these gene models were highly conserved and present many copies at different positions in the *Pp* genome. The fact that these genes have several copies in their genome indicates their decisive role in rusts pathogenicity, making evolutionary flexibility possible through enhanced allelic variation. This suggested that this family might have undergone expansion in the *Pp* genome or among other rust-causing species. All members in clusters VI, VII, and VIII in the phylogenetic tree (Table 1) are in an expanded family (4470) identified among 14 ruts and non-rust fungi described by Gupta et al., 2023, confirming our results.

In *Blumeria graminis* f. sp*. hordei* (Bgh), the multigene families Egh16 and Egh16H were identified (Grell et al., 2003). The authors suggested that those genes were restricted to filamentous fungi because no other organisms presented sequence homologies in the databases. The authors found multiple copies of Egh16-like genes (at least 10) in *B. graminis* encoding proteins with variable C-terminal regions implicated in mediating fungus-plant interactions. The presence of Egh16H in pathogenic fungi with necrotrophic, semi-biotrophic, and biotrophic lifestyles suggests its involvement in general fungal pathogenicity.

The Egh16-like virulence-related domain has been characterized in the pathogenic fungus *Erysiphe graminis* f. sp. *hordei* and plays a critical role in the early stages of infection (Grell et al., 2003). This domain has also been observed in effector candidates from *Puccinia graminis* f. sp. *tritici* and *Melampsora larici-populina* (Xue et al., 2002). However, its function in *Pp* and other rust fungi is poorly understood. The GAS1 protein in *Magnaporthe grisea*, which contains an Egh16-like domain, has been shown to participate in appressorial penetration and lesion formation, both of which are early events during infection (Xue *et al.*, 2002). The Egh16-like domain has been observed in transcripts highly expressed in nematode-trapping fungi (*Arthrobotrys oligospora*, *Monacrosporium cionopagum*, and *Arthrobotrys dactyloides*) during banana infections (Ma *et al.*, 2021). Recently, a potential function for the Egh16-like domain was shown in the pathogenic fungus *Metarhizium robertsii,* where Egh16-like proteins act as TLDD (Targeting Lipid Droplets for Degradation), promoting the generation of appressorium turgor for breaching host cuticles (Anderson et al., 2016). 

Likely, the function of the Egh16-like effectors in *Pp* is also involved in the initial stages of infection, given that the expression levels of family members were generally higher until 24 hai, decreasing afterward. The highest expression peak was observed at 12 hai (clusters VI and VII), which coincides with the development of *Pp* appressoria (Bonde et al., 1976). The highest expression induction observed for the transcript denovo_1784 and gene model correspondents (Cluster VI) at 12 hai may indicate the importance of this gene during the initial stages of the *Pp* infection process. The second most expressed gene, denovo_2238, and gene model correspondents (cluster VII) also showed a peak of expression at 12 hai.

Gene models corresponding to denovo_1784 and denovo_2238 strongly suppressed callose deposition and ROS production in *N. benthamiana* leaves. ROS production and callose deposition are considered basal defense responses associated with PTI and ETI. The production of ROS is caused by plant tissue injury and fungal or bacterial elicitors, resulting in the successive addition of electrons to molecular oxygen (O2), thereby generating molecules such as hydrogen peroxide (H2O2), which is associated with the induction of defense signaling in plants. Callose (1,3-β-glucan polymer) and lignin fortify the cell wall and act as an antimicrobial matrix (Buchanan et al., 2015). Suppression of callose deposition and ROS production following inoculation with recombinant EtHAn corroborates our hypothesis that Family 1/Egh16-like members function as *Pp* effectors. When not recognized, effector proteins inhibit the basal defense responses (PTI) and trigger effector susceptibility (ETS). There is no protocol for *P. pachyrhizi* transformation which could provide direct evidence for functions of its effectors. However, it is possible to use a bacterial type three secretion system for effector delivery to test for PTI and ETI suppression (Fabro et al., 2011). Previously, Kunjeti et al. (2016) showed PTI suppression for four *Pp* genes, and later, Qi et al. (2018) showed 17 *Pp*ECs (*P. pachyrhizi* effectors candidates) that suppress non-host plant immunity. *Pp*EC23 effector heterologous infiltration in *Nicotiana* resulted in PTI suppression, as well in soybean (Qi *et al.*, 2018).

The ability of denovo_2238 to suppress ETI has already been demonstrated (De Carvalho et al., 2017) using heterologous *Pseudomonas* pv*. tomato* (*Pst*) DC3000 type III secretion system (T3SS) that elicits ETI in *N. benthamiana* (Mysore and Ryu, 2004). Although ETI induction depends on R-Avr specific recognition and, therefore, requires tests on host R plants or genetic engineering for non-host R plant generation, PTI suppression is efficient for effector function validation, as it can reveal similar results in different plant species (Deb et al., 2018). 

In the current study, gene models identified in the reference genome MT2006 corresponding to two transcripts (denovo_1784 and denovo_2238) belonging to Family 1 described by De Carvalho et al. (2017) showed highly similar corresponding genomic sequences in the three genomes (MT2006, UFV02, and K8108). The essential effectors typically exhibit high conservation levels. In a comparative study of the rust species *Melampsora lini*, flax rust, poplar rust, wheat stem rust, and wheat stripe rust pathogens, the most probable identified effectors were highly conserved (Nemri et al., 2014), which is in line with the high conservation observed for *Pp* Family 1/Egh16-like effectors. Considering the monoclonal cycles of *Pp* uredospore multiplication in soybean fields, in the absence of sexual recombination and alternative hosts, single nucleotide polymorphisms (SNPs) may not be the most prevalent and significant source of variability in this fungus. Besides SNPs, copy number variations (CNV) promoting the gain or loss of (a)virulence genes are critical in diverse fungal species (Steenwyk and Rokas, 2018). Transposable elements (TEs) are present in more than 90% of the *Pp* genome (Gupta et al., 2023) and directly impact the variability and virulence profiles. The association of TEs with effectors is also influential because mutation rates can be increased if they are near TEs (Fouche et al., 2018). 

Based on the occurrence of Egh-16like members in three different genome assemblies containing 22, 18, and 18 members, we found evidence that these effectors are essential for virulence in *Pp* and are potentially part of the core effectors in this species. The ability of Pp Egh16-like members to suppress ETI (De Carvalho et al., 2017) and PTI (Figure 2) was described in an expanded family in the *Pp* genome, and the observation of induction of expression during early infection in soybean supports our hypothesis. Additional studies are necessary to confirm the targets in the host and demonstrate their importance in virulence. These effectors may be involved in the initial steps of infection and potentially in other mechanisms related to plant immunity. Our findings are important for elucidating the mechanisms underlying *Pp* pathogenicity and biology. Determining these mechanisms will support the development of effective control strategies.

## Supplementary material

The following online material is available for this article:

Figure S1 - Phylogenetic tree based on multiple sequence alignments between gene models of Egh16-like members from *P. pachyrhizi* from MT2006 genome correlated with members of the transcriptome.

Table S1 - Gene models correspondence to the Family 1 *de novo* transcripts in three genomes MT2006, PPUFV02 and K8180.

Table S2 - Characterization of *Pp* Family 1 gene models in the reference genomes of MT2006, PPUFV02, and K8108 isolates and its correspondence with *de novo* transcripts sequences (De Carvalho *et al.*, 2017).

Table S3 - Expression profile of the eight *Pp* effector candidates during interaction with soybean. The gene expression levels of the candidates in infected soybean leaves collected at 6, 12, 24 and 48 hours after inoculation (hai) are presented.
