# Supplementary material for: Variability and functional characterization of the Phakopsora pachyrhizi Egh16-like effectors
Source: Genet Mol Biol. 2024 Sep 2;47(3):e20230192. doi: 10.1590/1678-4685-GMB-2023-0192 (PMC11378017; doi:10.1590/1678-4685-GMB-2023-0192)
Supplement: Figure S1 - [file 1415-4757-GMB-47-03-e20230192-s1.pdf]

Genetics and Molecular Biology

DOI: https://doi.org/10.1590/1678-4685-GMB-2023-0192

Supplementary Material to "Variability and functional characterization of the *Phakopsora pachyrhizi* Egh16-like effectors"

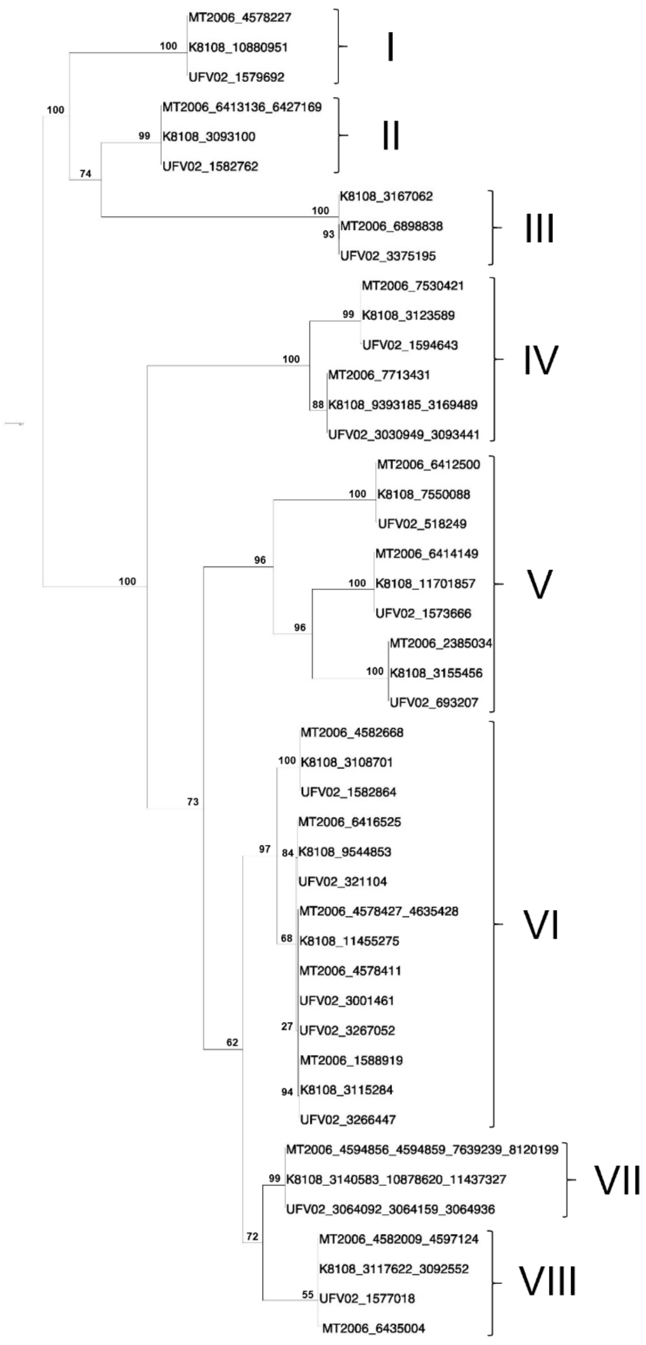

Figure S1 - Phylogenetic tree based on multiple sequence alignments between gene models of Egh16-like members from *P. pachyrhizi* from MT2006 genome correlated with members of the transcriptome.
