# Supplementary material for: Variability and functional characterization of the Phakopsora pachyrhizi Egh16-like effectors
Source: Genet Mol Biol. 2024 Sep 2;47(3):e20230192. doi: 10.1590/1678-4685-GMB-2023-0192 (PMC11378017; doi:10.1590/1678-4685-GMB-2023-0192)
Supplement: Table S3 - [file 1415-4757-GMB-47-03-e20230192-s4.pdf]

Genetics and Molecular Biology
DOI: https://doi.org/10.1590/1678-4685-GMB-2023-0192

Supplementary Material to "Variability and functional characterization of the *Phakopsora pachyrhizi* Egh16-like effectors"

Table S3 - Expression profile of the eight *Pp* effector candidates during interaction with soybean. The gene expression levels of the candidates in infected soybean leaves collected at 6, 12, 24 and 48 hours after inoculation (hai) are presented. Data obtained by RT-qPCR are presented as relative expression of the endogenous CytB gene.

**Potential genes analyzed by annealing of RT primers**	**Transcripts**	**Analyze at different infection times**				
		**Phylogenetic clade**	**6 hai**	**12 hai**	**24 hai**	**48 hai**
6413136 / 6427169	251	II	1,023	0,591	1,054	0,09
4578227	2595	I	1,147	1,01	1,064	0,152
4582009 / 4597124 / 6435004	555	VIII	0,709	1,001	0,772	0,037
6898838	635	III	0,991	1,113	***1,235*** *	***1,373*** *
4635428 / 4578427 / 4578411 / 4582668 / 6416525 / 7530421 / 7713431 / 1588919	1784	VI	2,136	***59,2*** *	***34,846*** *	***4,539*** *
8120199 / 7639239 / 4594856 / 4594859	2238	VII	1,252	***2,369*** *	0,696	0,41
6414149 / 6412500 / 2385034	6414149	V	1,108	1,426	1,018	0,173
7713431 / 7530421	7713431	IV	***1,602*** *	***1,563*** *	***2,885*** *	1,25

*Represents statistically significant differential expression.
